# Methylation and expression of glucocorticoid receptor exon-1 variants and FKBP5 in teenage suicide-completers

**DOI:** 10.1038/s41398-023-02345-1

**Published:** 2023-02-13

**Authors:** Hooriyah S. Rizavi, Omar S. Khan, Hui Zhang, Runa Bhaumik, Dennis R. Grayson, Ghanshyam N. Pandey

**Affiliations:** Department of Psychiatry, University of Illinois at Chicago, Chicago, IL 60612 USA

**Keywords:** Epigenetics and behaviour, Biomarkers

## Abstract

A dysregulated hypothalamic-pituitary-adrenal (HPA) axis has repeatedly been demonstrated to play a fundamental role in psychiatric disorders and suicide, yet the mechanisms underlying this dysregulation are not clear. Decreased expression of the glucocorticoid receptor (GR) gene, which is also susceptible to epigenetic modulation, is a strong indicator of impaired HPA axis control. In the context of teenage suicide-completers, we have systematically analyzed the 5’UTR of the GR gene to determine the expression levels of all GR exon-1 transcript variants and their epigenetic state. We also measured the expression and the epigenetic state of the FK506-binding protein 51 (FKBP5/FKBP51), an important modulator of GR activity. Furthermore, steady-state DNA methylation levels depend upon the interplay between enzymes that promote DNA methylation and demethylation activities, thus we analyzed DNA methyltransferases (DNMTs), ten-eleven translocation enzymes (TETs), and growth arrest- and DNA-damage-inducible proteins (GADD45). Focusing on both the prefrontal cortex (PFC) and hippocampus, our results show decreased expression in specific GR exon-1 variants and a strong correlation of DNA methylation changes with gene expression in the PFC. FKBP5 expression is also increased in both areas suggesting a decreased GR sensitivity to cortisol binding. We also identified aberrant expression of DNA methylating and demethylating enzymes in both brain regions. These findings enhance our understanding of the complex transcriptional regulation of GR, providing evidence of epigenetically mediated reprogramming of the GR gene, which could lead to possible epigenetic influences that result in lasting modifications underlying an individual’s overall HPA axis response and resilience to stress.

## Introduction

Suicide has been rising steadily for the past 10 years and is at its highest peak in the 30 years since data collection began [1]. This rise has occurred in every age group but is particularly high among teens 12–18 and college-age youth (2016 CDC WISQARS). Suicide is the result of a complex interplay between several factors, including age, sex, psychiatric disorders, neurobiological abnormalities, personality, and genetic and environmental factors [2–5]. From suicidal ideation, suicidal behavior, non-fatal suicide attempts, and finally, death, suicide is only one part of this continuum. Among environmental factors, there is considerable evidence that early life stress is associated with dysregulation in the hypothalamic-pituitary-adrenal (HPA) axis and can be a good predictor of suicide [6, 7]. Several studies have also shown that non-suppression of the dexamethasone suppression test (DST) is indicative of dysfunction of corticosteroid receptors, especially the glucocorticoid receptor (GR), and an impaired HPA axis [8, 9]. Evidence exists linking GR and suicidal behavior however, only a few studies directly measured the expression levels of GR in the postmortem brain of suicide-completers. For this purpose, Lopez et al. determined the mRNA expression of GR in the hippocampus but did not find a significant correlation between GR expression in suicide-completers compared to healthy controls [10]. In a subsequent study, Webster et al. found decreased levels of GR expression in the postmortem brain of patients with depression but not with suicide-completers [11].

**Fig. 1:**
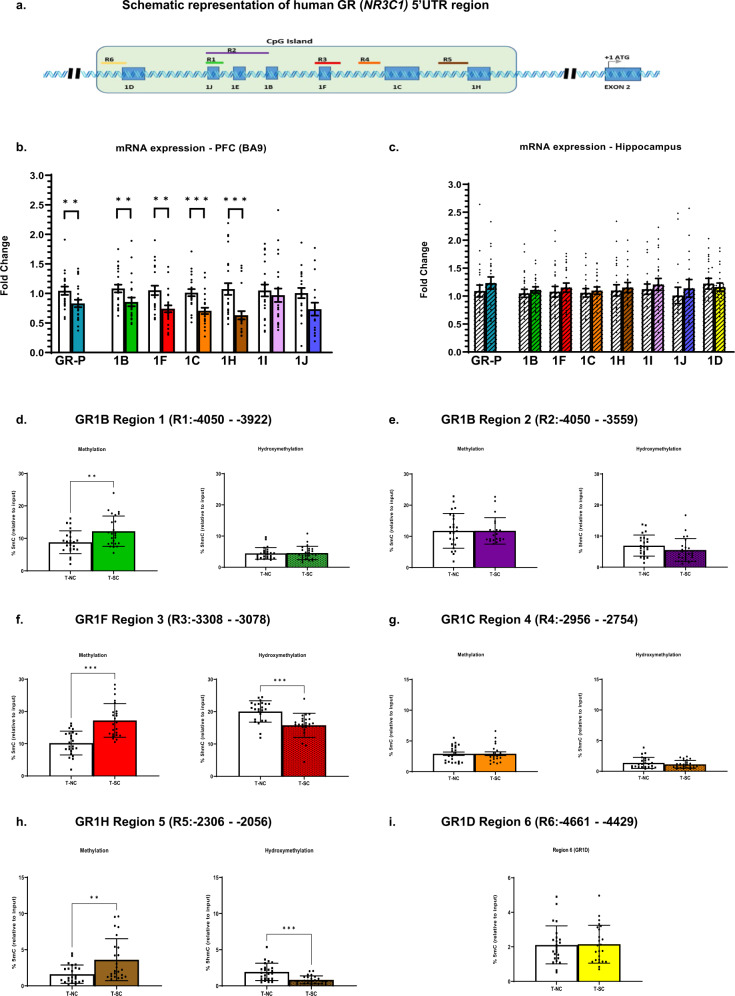
The human GR (*NR3C1)* 5’UTR region analyzed for expression, methylation (%5mC), and hydroxymethylation (%5hmC). **a** Schematic representation of human GR (*NR3C1)* 5’UTR region. Light green box illustrates CpG island with seven GR-1 transcript variants 1D-1H shaded in blue boxes and flags indicating expression primer location (common reverse primer located in exon 2). Regions analyzed for methylation and hydroxymethylation are labeled R1–R6. **b** Relative expression levels of GR-P and GR-1 transcript variants in PFC, GR-P (FC = 0.78, *p* < 0.01), GR-1B (FC = 0.86, *p* < 0.01), GR-1F (FC = 0.75, *p* < 0.01), GR-1C (FC = 0.71, *p* < 0.001), GR-1H (FC = 0.63, *p* < 0.001), GR-1E (FC = 1.05, *p* > 0.05), an d GR-1J (FC = 0.73, *p* > 0.05). White bars indicate normal-controls and color-filled bars indicate suicide-completers for each target gene. **c** Relative mRNA expression levels of GR-P and GR-1 transcript variants in hippocampus, GR-P (FC = 1.08, *p* > 0.05), GR-1B (FC = 1.11, *p* > 0.05), GR-1F (FC = 0.95, *p* > 0.05), GR-1C (FC = 0.97, *p* > 0.05), GR-1H (FC = 0.96, *p* > 0.05), GR-1E (FC = 0.95, *p* > 0.05), GR-1J (FC = 1.13, *p* > 0.05), GR-1D (FC = 1.03, *p* > 0.05). White bars indicate normal-controls and lined/color-filled bars indicate suicide-completers for each. Data are expressed as fold change (FC) ± SEM (error bars) after normalization to geometric mean of internal controls and suicide-completers compared to normal-control subjects. **d**–**i** In the PFC 5mC and 5hmC levels of 6 regions analyzed using meDIP and hmeDIP and presented as percent of input with respect to specific regions. **d**. R1 %5mC (*F* = 8.19, *p* = 0.01) levels and %5hmC (*F* = 0.029, *p* = 0.86) levels. **e** R2 %5mC (*F* = 1.37, *p* = 0.25) levels and 5%hmC (*F* = 1.80, *p* = 0.19) levels. **f**. R3 %5mC (*F* = 25.86, p < 0.001) levels and %5hmC (*F* = 18.86, *p* < 0.001) levels. **g** R4 %5mC (*F* = 0.07, *p* = 0.78) levels and %5hmC (*F* = 0.93, *p* = 0.34) levels. **h** R5 %5mC (*F* = 11.34, *p* = 0.002) and %5hmC (*F* = 18.43, *p* < 0.001) levels. **i** R6 %5mC (*F* = 0.01, *p* = 0.92). Results are expressed: %5mC or %5hmC relative to input ± SD (error bars) measured across each region (R1–R6) comparing suicide-completers (T-SC) to normal control (T-NC) subjects *N* = 24/24. *p < 0.05, **p < 0.01, ***p < 0.001.

The human GR gene (*NR3C1*) has a complex gene structure with multiple 5’ untranslated regions (5’UTR) consisting of nine non-coding exons/promoters, seven of which (1-B–1-H) reside in a CpG island (CGI) (Fig. 1). This CGI contains the recognition sequences for several transcription factors, multiple glucocorticoid response elements (GRE) and is subject to epigenetic influences through changes in DNA methylation. Initial studies reported by Weaver et al. [12] showed that insufficient maternal licking and grooming in rats results in the hypermethylation of GR exon-1_7_ (corresponding to GR-1F in humans), which, in turn, leads to the lack of Ngf1A binding and reduced GR transcription [12]. Similarly, McGowen et al. showed that the GR exon-1F promoter is hypermethylated in the hippocampus of suicide-completers who experienced abuse during childhood [13]. Subsequently, several lines of evidence indicate that epigenetic mechanisms regulate GR expression hence impacting the HPA axis [12, 13].

DNA methylation (5mC), catalyzed by DNA methyltransferases (DNMTs), has been the primary and most studied chemical modification of DNA [14, 15]. However, 5mC is reversible and undergoes dynamic modifications, including oxidation of 5mC forming 5-hydroxymethylcytosine (5hmC), a stable epigenetic mark and an intermediate in the active DNA demethylation pathway [14–16]. Active DNA demethylation is initiated by the ten-eleven-translocase (TET) family of dioxygenases, acting in concert with the growth arrest- and DNA-damage-inducible proteins GADD45 which interact with other epigenetic regulators and recruit DNA glycosylases and the base excision repair pathway (BER) [14–17].

In the context of teenage suicide-completers, here, we investigate changes in DNA methylation (5mC) and hydroxymethylation (5hmC) levels spanning the CGI of the NR3C1 gene and determine the RNA expression profiles of all variants expressed in this CGI. For FKBP5, we analyzed the RNA expression and 5mC and 5hmC levels of the promoter region covering a CGI and encompassing the transcription start site (TSS). In addition, we also measured the expression levels of DNA methylating (DNMT1, 3A, and 3B) and demethylating enzymes (TET1, 2, 3, and GADD45α, β, γ) as potential contributors to the altered epigenetic state. To our knowledge, no study has combined the examination of GR expression from a gene variant and methylation state perspective and a critical molecular regulator to determine which of these mechanisms are associated with teenage suicide-completers. Furthermore, our decision to study these mechanisms in the hippocampus and prefrontal cortex (PFC), Brodmann area 9 (BA9) in particular, is because of the involvement of these areas in many brain functions relevant to depression and suicide [18–20]. We provide convincing evidence that epigenetic changes may be driving the reprogramming of GR expression and regulation and thus contribute to an impaired HPA axis in teenage suicide-completers.

## Methods

Detailed method procedures, including complete demographic measures, qPCR, enrichment assays, and data analysis, are described in Supplementary Methods.

### Brain samples

Postmortem brain samples from 24 non-psychiatric healthy controls (normal-control) and 24 suicide-completers were obtained from Maryland Brain Collection at the Maryland Psychiatric Research Center, and the University of Illinois IRB approved all research. Brain tissue was collected only after a family member gave informed consent. A brief overview of cohort demographic measures is given in Table 1, and a more detailed description of postmortem diagnoses, method of suicide, medication at the time of death (Table S4) and brain dissection is given in Supplementary Methods.

**Table 1:** Teenage cohort demographic and statistical measures.

Demographic Measures	Suicide-completers (*n* = 24)	Normal controls (*n* = 24)	*p*-values	Effect size
Age in years average (range)	15.83 (12–20)	16.58 (13–19)	*t* = 1.33, *p* = 0.19	Cohen’s *D* = 0.38
Sex	15 Male	16 Male	*X*^2^ (1, *N* = 48) = 0.76	Phi = −0.04
	9 Female	8 Female		
Race	20 White 3 Black 1 Asian	11 White 13 Black	*X*^2^ (2, *N* = 48) = 0.007	Cramer’s *V* = 0.46
Antidepressants	20 No 4 Yes	24 No 0 Yes	NA	
Died of Hypoxia	11 No 13 Yes	24 No 0 Yes	NA	
Ethanol	19 No 5 Yes	24 No 0 Yes	NA	
RIN (PFC)	8.20 ± 1.29	8.34 ± 1.35	*t* = 0.37, *p* = 0.71	Cohen’s *D* = 0.11
RIN (HIPP)	5.82 ± 0.78	5.82 ± 0.90	*t* = −0.02, *p* = 0.99	Cohen’s *D* = 0
PMI	18.62 ± 7.04	18.54 ± 7.35	*t* = −0.04, *p* = 0.96	Cohen’s *D* = 0.01
Brain pH	6.17 ± 0.45	6.22 ± 0.39	*t* = 0.35, *p* = 0.73	Cohen’s *D* = 0.12

Cohen’s *D* of 0.38 indicates that the two group means differ by 0.38 standard deviations, Phi ranges from −1 to 1. Close to 1/−1 means a strong association between two variables. Cramer’s *V* ranges from 0 to 1, 1 represents a strong association and 0 represents no association.

*RIN* RNA integrity number, *PMI* postmortem interview, *PFC* prefrontal cortex, *HIPP* hippocampus.

### Transcript expression analysis

RNA was isolated using TRIZOL Reagent (Invitrogen) as per the manufacturers’ instructions. RNA quality was determined using an Agilent 2100 Bioanalyzer (Agilent Technologies). 1 µg of RNA was reverse transcribed using MMLV (Invitrogen) in a final reaction volume of 20 μl. Each PCR was carried out in duplicate, normalized to the geometric mean of ACTB and GAPDH, and expressed relative to normal-control samples using 2^−(ΔΔ*Ct*)^ method, where ΔC_t_ = Ct_target_−Ct_reference_ and ΔΔCt = Suicide-Subjects (Ct _target_–Ct _reference_)−(Ct_target_–Ct_reference_) [21, 22].

### DNA methylation enrichment assays

Changes in 5mC and 5hmC levels were determined using 1 μg of fragmented genomic DNA and immunoprecipitation (IP) using either an anti-5mC or anti-5hmC monoclonal mouse antibody included in the MeDIP or hMeDIP kit (Diagenode) according to the manufacturer’s instructions. qPCR was performed on immunoprecipitated and “input” DNA using custom primer pairs covering specific regions, and %5mC/%5hmC was calculated using the equation: % (Me/hMe DNA−IP/total input) = 2^[(Ct (10% input)−3.32)−Ct (MeDNA−IP)]×100.

### Data analysis

Statistical analyses were performed using the R statistical software package. For expression, a linear regression model was fitted to compare the effects of suicide-completers and normal-control subjects for all outcome variables separately. There are 7 GR exon-1 transcript variants (1B-1H), FKBP5, and 9 DNA methylating and demethylating enzymes (TET1, TET2, TET3, DNMT1, DNMT3a, DNMT3b, GADD45α, GADD45β, GADD45γ). The covariates included in the model were age, sex, race, PMI, brain pH, RIN, antidepressants, ethanol, and hypoxia. The final model included significant covariates only. All outcome variables were assumed normal based on Shapiro–Wilk test, and q–q plot. In addition to uncorrected p-values, FDR-adjusted *p*-values for multiple testing at 0.05 level are reported for GR transcripts and other target genes separately (see Supplemental Table S5). For DNA methylation, MANCOVA was used to compare the two groups for 5mC and 5hmC levels together across each region (R1–R6) separately. Covariates included in the model were age, sex, race, PMI, brain pH, antidepressants, ethanol, and hypoxia. Absence of multi-collinearity was performed for the independence of two outcome variables. Graphs are drawn using PRISM statistical analysis software (GraphPad, San Diego, CA). Correlation analysis was performed using Pearson’s correlation between 5mC, 5hmC, and qPCR data sets. All qPCR data are plotted as fold change (relative to control) ± SEM. Means of demographical variables—age, brain pH, RIN, and postmortem interval (PMI)—were compared using *t*-statistics whereas differences in race, and sex, for the 2 groups were analyzed using Pearson’s chi-square test (Table 1). For effect size calculation, Cohen’s *D* was reported for continuous variables (age, brain pH, RIN, and PMI), Phi Coefficient for sex, and Cramer’s *V* for race. Bartlett’s test of homogeneity of variances was conducted for between-group variation.

## Results

### Expression of GR exon-1 splice variants and GR-Pan (GR-P)

We performed qPCR analysis and determined that in the PFC, suicide-completers showed a significant decrease in mRNA levels corresponding to GR-P (*p* < 0.01), GR-1B (*p* < 0.01), GR-1F (*p* < 0.01), GR-1C (*p* < 0.001), and GR-1H (*p* < 0.001) when compared with normal-control subjects (Fig. 1b). We also analyzed the relative expression patterns of each variant in the PFC and hippocampus (Supplemental Fig. S2). Our results are consistent with previous reports where GR-1C is the most highly expressed variant, followed by GR-1B, GR-1H, and GR-1F [23, 24]. GR-1D was undetectable in PFC but is expressed in the hippocampus. Furthermore, the expression levels of GR-1E and 1J are relatively very low in both brain areas (Ct > 33) where within samples and group averages resulted in high standard deviations. For this reason, we chose not to include 1E and 1J in any further analyses. Unlike the PFC, the hippocampus showed no significant change in mRNA expression of GR-P and GR exon-1 variants (Fig. 1c). These results showed no significant effect of age, sex, race, PMI, brain pH, and RIN as determined by regression analysis. There is a significant effect of antidepressants on GR-P, however, after adjusting *p*-values for multiple comparisons, GR-P and GR exon-1 splice variants remained significant for the PFC (Supplemental Table S5)

### DNA methylation and hydroxymethylation analysis of GR CpG Island

To determine whether changes in mRNA levels of GR-1 variants corresponded with changes in 5mC and 5hmC levels at the promoter regions for specific GR-1 variants, we systematically examined six regions that span the CGI (Fig. 1a). Because we did not observe significant mRNA changes for the GR-1 variants in the hippocampus, we focused the methylation studies on the PFC and not the hippocampus.

For the GR-1B transcript, we examined two regions associated with this promoter [23, 25]. Denoted as R1 and R2, R1 has 9 CpGs, includes GR-1J transcript, and is upstream of GR-1E transcript (Fig. 1a). After adjusting for all covariates and multiple testing (FDR corrected) we found an increase in %5mC levels of R1 (*p* < 0.05) with no change in %5hmC levels (*p* = 0.91) (Fig. 1d). Interestingly, R2, which has 36 CpGs and overlaps with R1, shows no change in %5mC (*p* = 0.39) and %5hmC (*p* = 0.35) (Fig. 1e). R3 includes 22 CpGs, overlaps with the GR-1F promoter, and includes the non-canonical NGFI-A binding site. This region shows both a significant increase in %5mC (*p* < 0.001) and a significant decrease in %5hmC levels (*p* < 0.001) (Fig. 1f). R4, which includes 22 CpGs and is upstream of GR-1C promoter, showed no change in %5mC (*p* = 0.91) and %5hmC (*p* = 0.47) (Fig. 1g). R5 has 31 CpGs and is upstream of the GR-1H promoter. Here, we see a significant increase of %5mC levels (*p* < 0.01) and a significant decrease of %5hmC levels (*p* < 0.001) (Fig. 1h). Even though the mRNA levels of the GR-1D transcript were not detected in the PFC, we included a region immediately upstream of this exon, R6, as a control region. The levels of 5mC in R6 showed no differences (*p* = 0.91) (Fig. 1i), while 5hmC is undetectable in both groups in R6. Since the anti-5hmC antibody specifically recognizes 5hmC and not 5mC or unmodified DNA, the low or lack of amplification implies an absence of 5hmC in this region. All primer locations, the location of sequences amplified in each region relative to the start sites, and all corresponding *p*-values are provided in the Supplementary Methods.

### Correlation analysis of methylation and expression

**Fig. 2:**
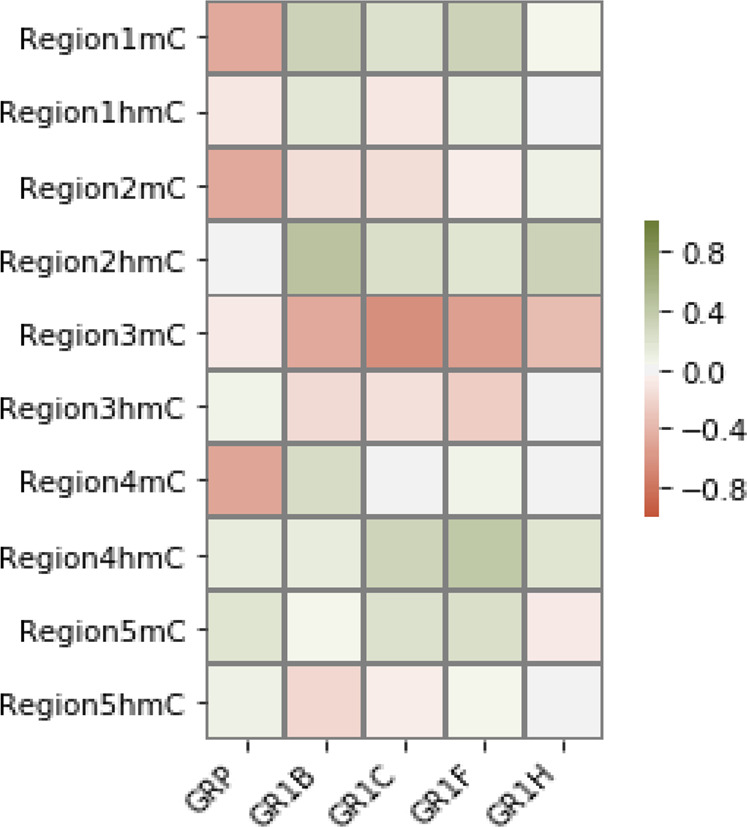
Heat map showing the correlation of methylation, hydroxymethylation, and expression of GR CpG island. Heat map represents the five regions (R1–R5) analyzed for changes in %5mC and %5hmC levels with expression data of GR-P and GR-1 variants (B–H). Red cells signify an increase in 5mC and down-regulated expression, green cells signify an increase in 5hmC and up-regulated expression. Correlation analysis was performed using Pearson’s correlation based on %5mC, %5hmC values, and fold change in log_2_ values of gene expression data.

We next correlated the methylation (%5mC) and hydroxymethylation (%5hmC) changes with mRNA expression to determine whether significant positive and/or negative correlations were observed in the levels of methylation in each region compared to the expression of GR-P or selected GR-1 variants (Fig. 2). A strong negative correlation was observed between GR-P mRNA and %5mC of R1 (*r* = −0.47, *p* = 0.02), %5mC of R2 (*r* = −0.46, *p* = 0.02), and %5mC of R4 (*r* = −0.48, *p* = 0.02). There is a strong negative correlation between %5mC of R3 and mRNA of GR-1B (*r* = −0.47, *p* = 0.02), GR-1C (*r* = *−*0.69, *p* < 0.01), and GR-1F (*r* = −0.53, *p* < 0.01) but not with GR-P and GR-1H. There is also a strong positive correlation between %5hmC of R2 and mRNA of GR-1B (*r* = 0.46, *p* = 0.02) and between %5hmC of R4 and mRNA of GR-1F (*r* = 0.41, *p* = 0.02). Interestingly, the significant decrease in %5hmC levels of R3 did not correlate with mRNA of GR-P or GR-1F. One possibility could be because of the overall low amounts of 5hmC and the variability in each sample. Of note, all regions show much lower levels of 5hmC compared to corresponding 5mC levels, except R3 and R5 which showed approximately equal detection levels. R4 has an overall low level of methylation compared to other regions (Fig. 1d–h).

### Expression and methylation analysis of FKBP5

**Fig. 3:**
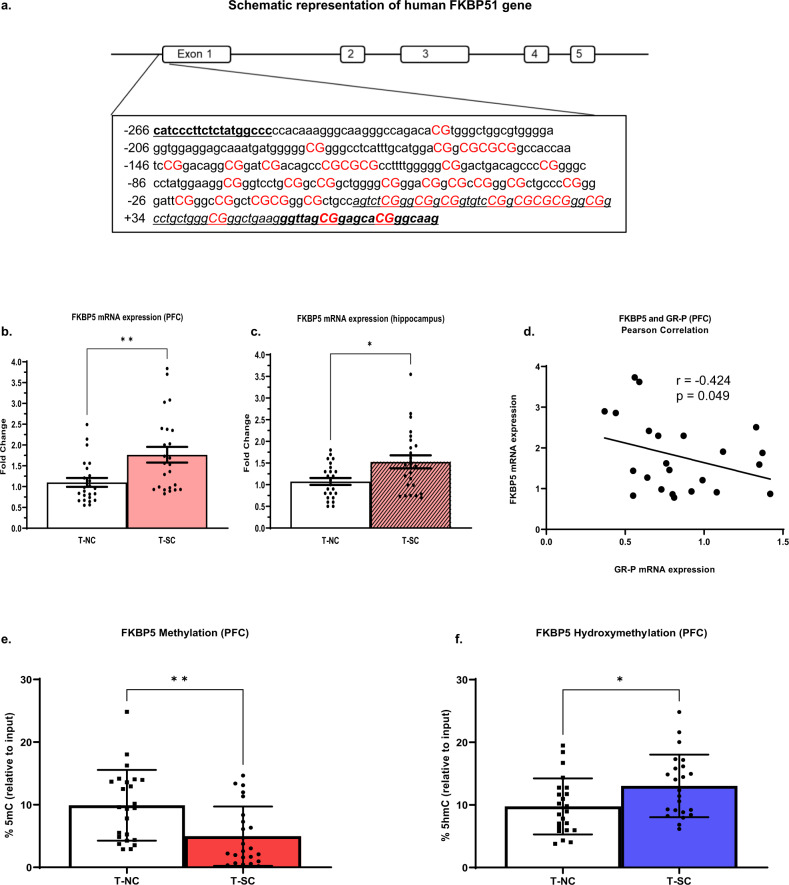
Human FKBP5 mRNA expression, methylation, and hydroxymethylation analysis. **a** Schematic representation of the human FKBP51 gene proximal promoter region indicating Transcription start site (TSS). The analyzed region −266 to +73 (340 bp) which includes 39 CpGs (depicted in red uppercase letters). The numbering is relative to the TSS. Underlined/italicized letters indicate the exon, and bold/black letters indicate primers used for meDIP/hmeDIP-qPCR analysis. **b** Relative expression levels of FKBP5 mRNA in PFC (FC = 1.61, *p* = 0.0009), and **c** hippocampus (FC = 1.36, *p* = 0.02). Data are expressed as fold change (FC) ± SEM (error bars) after normalization to the geometric mean of internal controls and suicide-completers compared to normal control subjects. **d** Pearson’s correlation analysis of FKBP5 and GR-P mRNA levels in the PFC. **e** and **f** In the PFC, 5mC, and 5hmC levels across the TSS region of FKBP5 gene analyzed using meDIP and hmeDIP and presented as percent of input. **e** %5mC levels (*t* = 3.22, *p* = 0.002) and **f** %5hmC (*t* = −0.25, *p* = 0.02). Results are expressed: %(5mC/5hmC) relative to input ± SD (error bars) measured across each region comparing suicide-completers (T-SC) to normal-control (T-NC) subjects. *N* = 24/24. *p < 0.05, **p < 0.01.

To further probe the regulation of GR, we examined the mRNA expression of FKBP5. In suicide-completers, FKBP5 mRNA is significantly increased in the PFC (*p* < 0.01) and hippocampus (*p* < 0.05) (Fig. 3b, c). Interestingly, we observe a strong inverse correlation between GR-P mRNA and FKBP5 mRNA (*r* = −0.424, *p* = 0.049) (Fig. 3d). To determine if this increase in FKBP5 mRNA results from an altered methylation state, we analyzed a locus directly upstream and overlapping the TSS (−245 to +68) (Fig. 3a) of *FKBP5*. This region shows a significant decrease in %5mC (*p* < 0.01) and an increase in %5hmC levels (*p* < 0.05) (Fig. 3e, f). This shift in DNA methylation levels may result in the observed increase in FKBP5 mRNA, causing a further impediment to GR signaling and HPA axis dysregulation. Age is a significant covariate in the PFC however, FDR-corrected *p*-values for multiple comparisons for FKBP5 mRNA, %5mC, %5hmC levels remained significant (Supplemental Table S5).

### Expression of DNA modifying enzymes

**Fig. 4:**
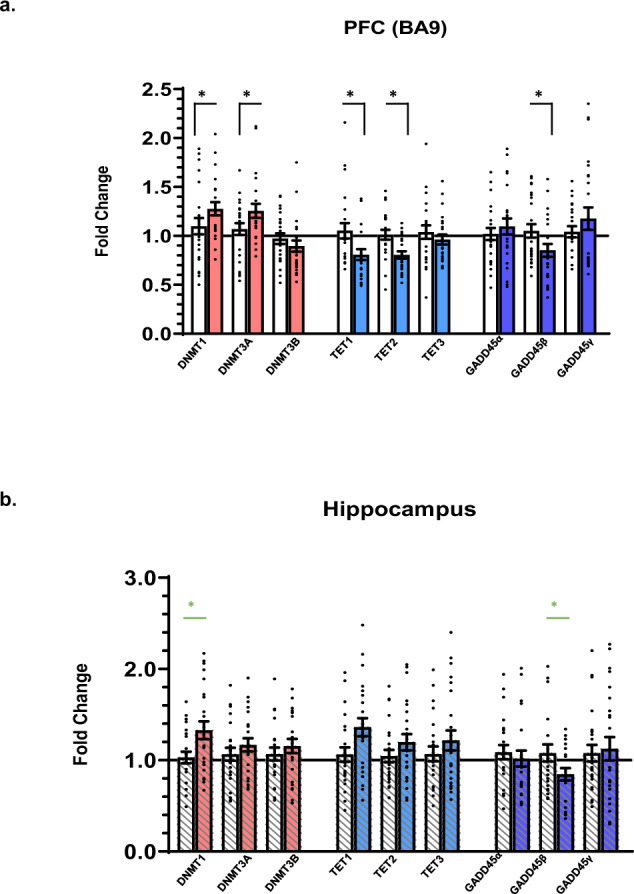
Relative expression levels of DNA methylating and DNA demethylating enzymes. **a** Relative mRNA expression levels in the PFC of DNA methylating enzymes: DNMT1 (FC = 1.34, *p* = 0.02), DNMT3A (FC = 1.25, *p* = 0.02), DNMT3B (FC = 0.86, *p* = 0.11), DNA demethylating enzymes: TET1 (FC = 0.72, *p* = 0.03), TET2 (FC = 0.81, *p* = 0.02), TET3 (FC = 0.96, p = 0.52), GADD45α (FC = 1.05, *p* = 0.43), GADD45β (FC = 0.86, *p* = 0.03), GADD45γ (FC = 1.05, *p* = 0.81). **b** In hippocampus: DNMT1 (FC = 1.27, *p* = 0.02), DNMT3A (FC = 1.10, *p* = 0.29), DNMT3B (FC = 1.07, *p* = 0.48), DNA demethylating enzymes: TET1 (FC = 1.28, *p* = 0.08), TET2 (FC = 1.12, *p* = 0.25), TET3 (FC = 1.11, *p* = 0.34), GADD45α (FC = 0.92, *p* = 0.44), GADD45β (FC = 0.78, *p* = 0.04), GADD45γ (FC = 0.96, *p* = 0.6). White bars indicate normal-controls and lined/color-filled bars indicate suicide-completers for each target gene. Data are expressed as fold change (FC) ± SEM (error bars) after normalization to the geometric mean of internal controls and suicide-completers (T-SC) to normal-control (T-NC) subjects. *N* = 24/24, *p < 0.05, green *p indicates FDR corrected *p* is not significant.

Next, we sought to investigate the expression levels of enzymes necessary for maintaining the dynamic balance between DNA methylation and demethylation in both brain regions. We determined the mRNA levels of DNA methyltransferases (DNMT-1, -3A, -3B) as they are the primary contributors to the DNA methylation pathway. We also analyzed Ten-Eleven-Translocation protein family members (TET-1, -2, -3) and Growth Arrest and DNA Damage 45 (GADD45-α, -β, -γ) mRNAs as key participants in the DNA demethylation cascade. In suicide-completers (see Fig. 4a and b), DNMT1 and DNMT3A mRNA levels are increased in the PFC (*p* < 0.05), and (*p* < 0.05) respectively. DNMT1 is also increased in the hippocampus (*p* = 0.02), however, after correcting for multiple comparisons this change is no longer significant (Fig. 4b) FDR *p* > 0.05 (Supplemental Table S5). The expression of DNMT3B showed no significant change in both brain areas. PMI is a significate covariate for DNMT3B in the PFC. TET1 and TET2 mRNA levels are significantly decreased (*p* < 0.05) and (*p* < 0.05) respectively, while TET3 shows no change in both brain areas. Antidepressant is a significant covariate in the PFC for TET1.

Members of the GADD45 gene family are involved in active DNA demethylation, particularly in the nervous system and in response to genotoxic and physiological stress [26]. In suicide-completers, GADD45β mRNA is decreased in the PFC (*p* < 0.05) and although initial analysis showed a decrease in hippocampus (*p* < 0.05) (Fig. 4b), after correcting for multiple comparisons this change is no longer significant (FDR *p* > 0.05) see Supplemental Table S5. GADD45α and GADD45γ mRNAs remained unchanged in both brain areas. Of note, in the PFC our results indicate that DNA methylating and demethylating enzymes are aberrantly expressed, which very likely contributes to both global and locus-specific DNA methylation states. DNMT1, DNMT3A, TET1, TET2, and GADD45β remained significant in the PFC (Fig. 4a). In the hippocampus, all initial significant observations are no longer significant after multiple testing corrections (Supplemental Table S5).

## Discussion

To investigate possible mechanisms driving GR dysregulation in teenage suicide-completers, we combined three different types of measurements: mRNA levels of distinct promoters corresponding to the 5’ exon of *NR3C1*, methylation status (either 5mC or 5hmC) at specific sites along with the multiple, independent promoter/non-coding exons present in the CpG island that defines the 5’end of *NR3C1*, and mRNAs encoding enzymes operative in defining the site-specific balance between DNA methylation and demethylation pathways. Here, we report changes in both 5mC and 5hmC levels at specific regions in the CGI of the GR and expression of specific GR-1 non-coding variants.

The 5’ end of the GR gene is highly complex and is transcriptionally regulated by several untranslated exon-1 variants. Moreover, these non-coding variants (Fig. 1) are susceptible to regulation by changes in DNA methylation. The six regions analyzed for DNA methylation (R1–R6) correspond to distinct promoter regions of the seven non-coding first exons. Methylation changes at specific sites have been associated with a lack of transcription-factor (TF) binding, and the GR CGI contains several putative TF binding sites. These include recognition sites for SP-1 and Yin-Yang 1 (YY1) located in the promoter regions of 1B and 1C, AP-1 and AP-2 sites in promoter 1C, and most notably, the canonical binding site for NGF1-A located in promoter 1F [27–29]. Although recent studies have elucidated some of the functional consequences of variations in methylation of GR-promoters relevant to the stress response, these are primarily related to the consequence of early life adversity (ELA) and mainly occur in peripheral tissues [30–35]. Consistently, these studies show an increase in GR-1F methylation leading to downregulation of GR mRNA in these subjects [36]. McGowan et al. reported increased GR-1F promoter methylation with decreased GR mRNA in the hippocampus of adult suicide-completers with a history of ELA [13]. Labonte analyzed GR-1D, 1C, and 1H and reported hypermethylation of specific CpGs at promoter/exons 1B and 1C that negatively correlated with expression of 1B and 1C mRNAs. Hypomethylation of 1H was positively correlated with 1H expression in the hippocampus of suicide-completers with ELA [23]. While these changes were reported only in the hippocampus of adult suicide-completers with ELA, in our cohort, with no documented history of ELA, we show that the expression of GR-1B, -1C, -1F, and -1H are decreased in the PFC. In contrast, the hippocampus exhibited no detectable changes. Additionally, we observed a significant increase in 5mC content in R1, R3, and R5 accompanied by a loss in 5hmC content in R3 and R5, which in some GR-1 variants are reflected in the corresponding changes in mRNA expression. There is a strong negative correlation between R3 5mC and the expression of 1B, 1C, and 1F. Also, 5hmC levels at R2 and R4 are positively correlated with the expression of 1B and 1F, respectively. The noted changes in DNA methylation likely drive these alterations at specific loci, resulting in a shift of alternative exon usage, affecting alternative splicing and expression of GR.

We further show a significant decrease in 5mC levels and an increase in 5hmC levels proximal to the TSS region of FKBP5, suggesting an epigenetic shift resulting in increased expression, which we observed in both PFC and hippocampus. The significance of changes in 5mC and 5hmC in relation to the GR variants is not clear. There may be changes in gene expression without concomitant changes in 5mC and 5hmC levels for GR variants. The roles of 5mC, and particularly of 5hmC, are not completely understood. One of the arguments could be that 5mC facilitates gene repression while 5hmC promotes gene expression in proximity to where the changes are taking place. Several studies have demonstrated the association of childhood trauma and epigenetic changes in FKBP5 with the development of major depression, PTSD, anxiety, and suicide [37–40]. For example, in one study, the frontal cortex of individuals with major depression shows increased FKBP5 expression while no change is observed with childhood maltreatment [41, 42]. Another interesting study reported that FKBP5 protein and mRNA are significantly decreased in the amygdala of undiagnosed and untreated suicide-completers [43]. Together, these findings indicate that variations in *FKBP5* are associated with mood disorders and suicidal behaviors.

In addition to variability at the 5’end, alternative splicing of the 3’end of the GR gene generates three main protein isoforms, GRα, GRβ, and GR pan (GR-P), each thought to be functionally distinct [44]. We have previously reported a decrease in the expression of GRα in PFC (*p* < 0.05) with no significant change in the hippocampus, and GRβ expression was undetected in this cohort [45]. Here, we determined the expression of the third GR isoform, GR-P, which consists of 676 amino acids and lacks exons 8 and 9 that encode the carboxyl-terminal half of the ligand binding domain and does not bind GCs [44, 46]. Although not much is known functionally about this isoform, in specific cell types, co-expression of GR-P with GRα leads to a more active form of GRα, resulting in enhanced GC bioactivity [46–49].

Another significant finding of this study is the dysregulation of enzymes involved in maintaining the dynamic status of DNA methylation/hydroxymethylation in both the PFC and hippocampus. Both DNMT1 and 3A are abundantly expressed in the brain. Thus, it is interesting that we see an increase of DNMT1 and DNMT3A in the PFC which likely leads to an overall increase in DNA methylation. For proteins involved in the DNA demethylation pathway, TET1, TET2, and GADD45β are significantly decreased in the PFC. GADD45β has been shown to regulate activity-dependent DNA demethylation at specific loci in genes like BDNF and fibroblast growth factor 1 (Fgf1) that have been implicated in neurogenesis, synaptic plasticity, and psychosis [50–53]. Notably, 5hmC is enriched in the brain and is associated with regulating neuronal activity [54, 55]. Furthermore, numerous studies have shown genome-wide disruptions in 5hmC levels associated with anxiety, stress-induced behaviors, and psychiatric illnesses, including depression and schizophrenia [56–58]. Collectively, this suggests that, at least in the PFC, there may be widespread increases in repressive programming resulting in decreased expression of numerous genes regulating stress susceptibility.

This study adds to the growing evidence linking epigenetic influences and disease outcomes. It shows that in teenage suicide-completers, epigenetic reprogramming of DNA methylation, may contribute to the aberrant expression profile of genes underlying the HPA axis dysregulation, which likely decreases resilience to the adaptive stress response and increases susceptibility to psychiatric disorders and suicide. There is significant evidence from human studies that childhood maltreatment is associated with altered brain development, specifically in the fronto-limbic circuitry [59–62]. This fronto-limbic circuitry is also enriched in GRs and is affected by chronic exposure to GCs [63, 64]. Indeed, epigenetic modifications regulate GR expression levels, influence HPA function, and affect other signaling pathways involved in the neurodevelopment of suicide [65–68]. The hippocampus is a critical area in inhibiting further activation of the HPA axis. Therefore, it is interesting that we did not find any statistically significant changes in the hippocampus. However, the brain is still developing during the teenage years, which makes it difficult to interpret these findings directly.

Suicide in general, and suicide in adolescence represents a major public health problem as it results in a large loss of life among teenagers and is probably the second highest cause of death among adolescents. The current study provides novel information regarding epigenetic mechanisms operative in teenage suicide PFC and hippocampus. However, there are several limitations to our study. One is the relatively small sample size (i.e., 24 teenage suicide subjects and 24 teenage control subjects), and another is the fact that the suicide cohort consists of several diagnostic groups, including major depression, alcohol abuse, drug abuse, and conduct disorders. This resulted in smaller groups of uniform diagnoses and limited power for testing diagnostic specificity. Another limitation of the study is that some of the suicide subjects were treated with psychoactive drugs at the time of death. In addition, the smaller sample size of the cohort limits the utility of genome-wide studies of DNA methylation, which require large sample sizes to obtain meaningful data. Finally, we were unable to determine whether the changes we observed were the consequence of alterations in the distribution of cell types present in the PFC. Given the above limitations, the present study is still relevant as it provides useful data regarding the expression and epigenetics of GR variants and chaperone proteins.

## Supplementary information

Supplementary Methods and Figures

## Data Availability

All code written in support of this publication is publicly available at https://github.com/rbhaumik/Mythylation-and-expressions-in-suicide.
