# Supplementary material for: Methylation and expression of glucocorticoid receptor exon-1 variants and FKBP5 in teenage suicide-completers
Source: Transl Psychiatry. 2023 Feb 13;13:53. doi: 10.1038/s41398-023-02345-1 (PMC9925759; doi:10.1038/s41398-023-02345-1)
Supplement: Supplementary file 1 — Supplementary Methods and Figures [file 41398_2023_2345_MOESM1_ESM.docx]

**Supplementary Methods**

**Acquisition of human postmortem brain samples**

Brain tissue samples were obtained from the Maryland Brain Collection at the Maryland Psychiatric Research Center, Baltimore, Maryland, in collaboration with the Office of the Chief Medical Examiner of the State of Maryland. Tissue samples were obtained from 24 teenage suicide victims and from 24 teenage control subjects (Table 1 and Table S4). Toxicological data were obtained by analysis of urine and blood samples from these subjects.

Samples were collected after informed written consent and detailed interview of at least one family member of the deceased. The interviews were conducted by trained psychiatrist and brain tissue from all subjects was thoroughly examined by experienced neuropathologists. Blood and urine samples were used to access the toxicology details. All procedures were approved by the University of Maryland Institutional Review Board (IRB) and by the University of Illinois at Chicago IRB.

The study was performed using frozen postmortem brain samples from prefrontal cortex [(PFC), Brodmann area 9 (BA9)] and the hippocampus head from the left hemisphere. We received already dissected tissue samples from the Maryland Brain Collection.

**Postmortem diagnostic procedure**

All subjects in this study were diagnosed using the Diagnostic Evaluation After Death (DEAD) (Salzman et al., Diagnostic Evaluation After Death [DEAD]. Rockville, MD: National Institute of Mental Health, Neuroscience Research Branch, 1983). and the Schedule for Clinical Interviews for the DSM-IV (SCID) (First et al., Structured Clinical Interview for DSM-IV Axis I Disorders (SCID-I). Arlington, Virginia, USA: American Psychiatric Publishing, Inc., 1997). The SCID was administered by a trained interviewer using a family member as an informant and included a review of all obtainable medical and psychiatric records. The DEAD is used only as a data organization instrument. The SCID diagnoses are validated by two trained psychiatrists. This has been found to be a very accurate way to make diagnoses (Ramirez Basco et al., Methods to improve diagnostic accuracy in a community mental health setting. The American Journal of Psychiatry 157, 1599-1605, 2000).

Family members gave permission for the use of brain tissue for research and for clinical records to be obtained from mental health treatment providers when there was a prior history of mental health treatment, or suicide. Two senior psychiatrists provided independent DSM-IV diagnoses. Consensus diagnostic report ensured that control subjects had no mental illness. The subjects with a history of substance abuse, family history of psychiatric illness, accidental deaths or death after prolonged hospitalization were excluded from the control group. Additionally, none of the subjects with any other mental illness, major history of medical or neurological disorders and HIV were included in any of the study groups. All subjects were considered without any sexual or ethnic differentiation. Detailed characteristics of the subjects of all three groups are listed in Supplementary Table S4.

**Brain tissue dissection procedure**

The postmortem brain samples from different brain regions were received from the Maryland Brain Collection at the Maryland Psychiatric Research Center already dissected. Brains were either stored in heat sealed bags in a -80°C freezer or dissected fresh. All frozen tissue was dissected with a Stryker autopsy saw. The prefrontal cortex was dissected and the position of BA9 is indicated in Fig. S1. The hippocampus and amygdala were removed together with the underlying entorhinal cortex. The hippocampus was divided into “head” and “tail” portions. For the mRNA studies we used the head of the hippocampus from the left hemisphere.


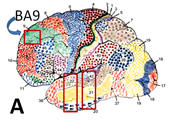


**Figure S1:**

**Primer design**

For the different non-coding GR exon 1 mRNA transcript variants, forward primers were designed to span the specific exon 1 variant and part of the common expressed exon 2, keeping the reverse primer for all variants the same (Table S1). In addition, all primer pairs were optimized for annealing temperature and concentration. Pre-designed TaqMan gene expression assays (Applied Biosystems) were used (Table S2) for expression analysis of all the remaining target genes. Twelve internal control genes were assayed using the geNORM (PrimerDesign Ltd) program according to the manufacturer's instructions for comparison before selecting ACTB and GAPDH as the most stable genes for normalization in our cohort.

**RNA isolation and reverse transcription**

100mg frozen tissue from PFC(BA9) and hippocampus was used to isolate RNA with TRIZOL Reagent (Invitrogen) followed by DNase I (Ambion) treatment to eliminate any trace amount of DNA. All RNA is stored at -80°C until use. Reverse transcription (RT) for each sample was performed using 1 μg total RNA, 200U MMLV-reverse transcriptase (Invitrogen), 50 ng Random Hexamers (Invitrogen), 2 mM dNTP mix (Invitrogen), 10U RNaseOUT (Invitrogen) in a 20 μl total volume and cycling conditions were carried out as per manufactures' instructions. After completion, cDNA is diluted 1:10 fold and stored at -20°C.

**Real-Time Polymerase Chain Reaction (qPCR)**

Each qPCR reaction contained 5μl template cDNA (1:10 dilution of RT), 10μl Power SYBR green (for custom primers) / TaqMan gene expression (TaqMan assays) master mix (2X) (Applied Biosystems), 1μl custom primer mix (20 μM) or pre-designed TaqMan assay (20 μM) in a total volume of 20 μl. The qPCR efficiency was tested over a series of 5-log dilutions to confirm that target and reference genes had similar amplification efficiencies. Each qPCR plate includes a "no reverse transcriptase" and "no template" control to verify non-specific amplification and primer-dimer, respectively. To calculate relative expression, replicate mean values of target gene Ct is normalized to the geometric mean of reference genes, ACTB and GAPDH, and is expressed relative to the control samples using 2^−(ΔΔ^*^Ct^*^)^ method, where ΔΔCt = (Ct _target_ - Ct _reference_) Suicide Subjects - (Ct _target_ - Ct _reference_) Normal Controls. Outliers were excluded if the normalized ΔC_t_ values were greater than 2 standard deviations from the group mean. QPCR product for each primer pair was confirmed by gel electrophoresis, and products from custom-designed primers were also analyzed by monitoring the dissociation curve. Relative expression levels are plotted as fold change, and ΔC_t_ values are used for further statistical analysis.

**DNA isolation**

Genomic DNA (gDNA) was isolated from 50mg of frozen tissue, PFC(BA9), using DNeasy Blood and Tissue Kit (Qiagen) following the manufactures' instructions. The quality of gDNA was assessed and quantified with the Qubit™ dsDNA Broad Range Assay Kit (ThermoFisher Scientific), and gDNA was stored at −20 °C until use.

**DNA methylation enrichment assays: methylated and hydroxymethylated DNA immunoprecipitation**

Genomic DNA was diluted to 0.1 μg/μl and fragmented to a mean size of approximately 250 bp, using the Bioruptor, and analyzed on an agarose gel before immunoprecipitation (IP). Methylated DNA (MeD) and hydroxymethylated DNA (hMeD) IP was performed using the MeDIP and hMeDIP kits (Diagenode, NJ, USA) according to the manufacturer's instructions. Briefly, all enrichments are performed on 1μg of fragmented gDNA spiked with internal positive (MeD/hMeD) and negative (unMeD/unhMeD) DNA controls and incubated in an IP mix overnight with anti-5mC/anti-5hmC monoclonal mouse antibodies. Each sample was IP'd in triplicates, and "input" samples were processed in parallel. %5mC/%5hmC quantification was performed on IP'd and input DNA and were normalized with the spike-in control (MeD/hMeD) for IP efficiency. Control primers included were TSH2B (meD), Sfi 1 (hMeD), and GAPDH (unMeD). Custom primers were used for the six regions analyzed (Table S3).

**
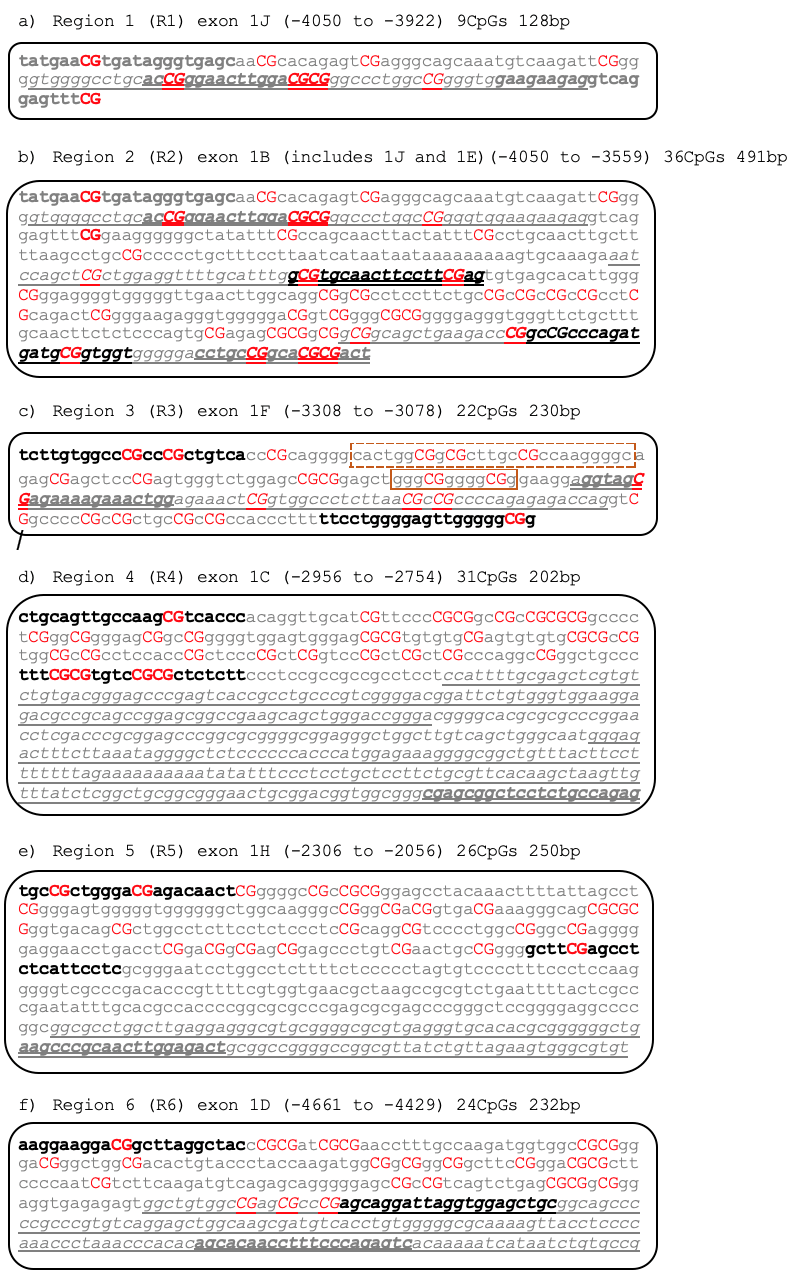
**

GR 5' UTR region, sections a-f represent the promoter regions analyzed (R1-R6) for methylation changes and primer sequences used for meDIP/hmeDIP-qPCR with amplicon size noted. The numbering is relative to the translation start site located in exon 2. The number of CpGs included in each region are noted and depicted in red with uppercase letters. Underlined/italicized letters indicate the exon, bold/black letters indicate primers used for meDIP/hmeDIP-qPCR, double-underlined letters indicate expression primer for that exon. CpGs included in the amplified region 3 (GR-1F) are CpGs #32-#53, solid-lined box is putative NGFI-A binding site and broken-lined box is non-canonical NGFI-A binding site as noted by McGowan et al. 2009.

**Table S1. Custom designed primer sequences for qPCR expression analysis of GR exon-1 variants**

| **Gene** | **Forward Primer Sequence** | **Reverse Primer Sequence** |
| --- | --- | --- |
| *GR-1B | cctgccggcacgcgact | cagtggatgctgaactcttgg |
| GR-1C | cgagcggctcctctgccagag | cagtggatgctgaactcttgg |
| *GR-1D | agcacaacctttcccagagtc | cagtggatgctgaactcttgg |
| *GR-1E | gcgtgcaacttccttcgag | cagtggatgctgaactcttgg |
| *GR-1F | aggtagcgagaaaagaaactgg | cagtggatgctgaactcttgg |
| *GR-1H | aagcccgcaacttggagact | cagtggatgctgaactcttgg |
| GR-1J | accgggaacttggacgcg | cagtggatgctgaactcttgg |

*1-3 base pairs upstream of primers from Turner et al., 2005

**Table S2. Pre-designed TaqMan primer/probe product codes used for gene expression analysis**

| **Gene** | **Taqman Accession** | **Probe Location (exon boundary)** | **Assay Function** |
| --- | --- | --- | --- |
| ACTB | Hs99999903_m1 | 1-1 | House Keeping (HK) |
| GAPDH | Hs99999905_m1 | 3-3 | HK |
| GR-P | Hs00230818_m1 | 7-8 | target gene |
| FKBP5 | Hs01561006_m1 | 6-7 | target gene |
| DNMT1 | Hs00945875_m1 | 17-18 | target gene |
| DNMT3A | Hs01027166_m1 | 15-16 | target gene |
| DNMT3B | Hs00171876_m1 | 6-7 | target gene |
| TET1 | Hs00286756_m1 | 11-12 | target gene |
| TET2 | Hs00325999_m1 | 9-10 | target gene |
| TET3 | Hs00379125_m1 | 9-10 | target gene |
| GADD45α | Hs00169255_m1 | 2-3 | target gene |
| GADD45β | Hs00169587_m1 | 2-3 | target gene |
| GADD45γ | Hs02566147_s1 | 4-4 | target gene |

**Table S3. Custom designed primers sequences used for methylation analysis of GR proximal promoter region**

|  | **Location** | **Forward Primer Sequence** | **Reverse Primer Sequence** |
| --- | --- | --- | --- |
| **Region 1** | -4050 to -3922 | tatgaacgtgatagggtgagc | gaagaagaggtcaggagtttcg |
| **Region 2** | -4050 to -3559 | tatgaacgtgatagggtgagc | gccgcccagatgatgcggtggt |
| **Region 3** | -3308 to -3078 | tcttgtggcccgcccgctgtca | ttcctggggagttgggggcgg |
| **Region 4** | -2956 to -2754 | ctgcagttgccaagcgtcaccc | tttcgcgtgtccgcgctctctt |
| **Region 5** | -2306 to -2056 | tgccgctgggacgagacaact | gcttcgagcctctcattcctc |
| **Region 6** | -4661 to -4429 | aaggaaggacggcttaggctac | agcaggattaggtggagctgc |


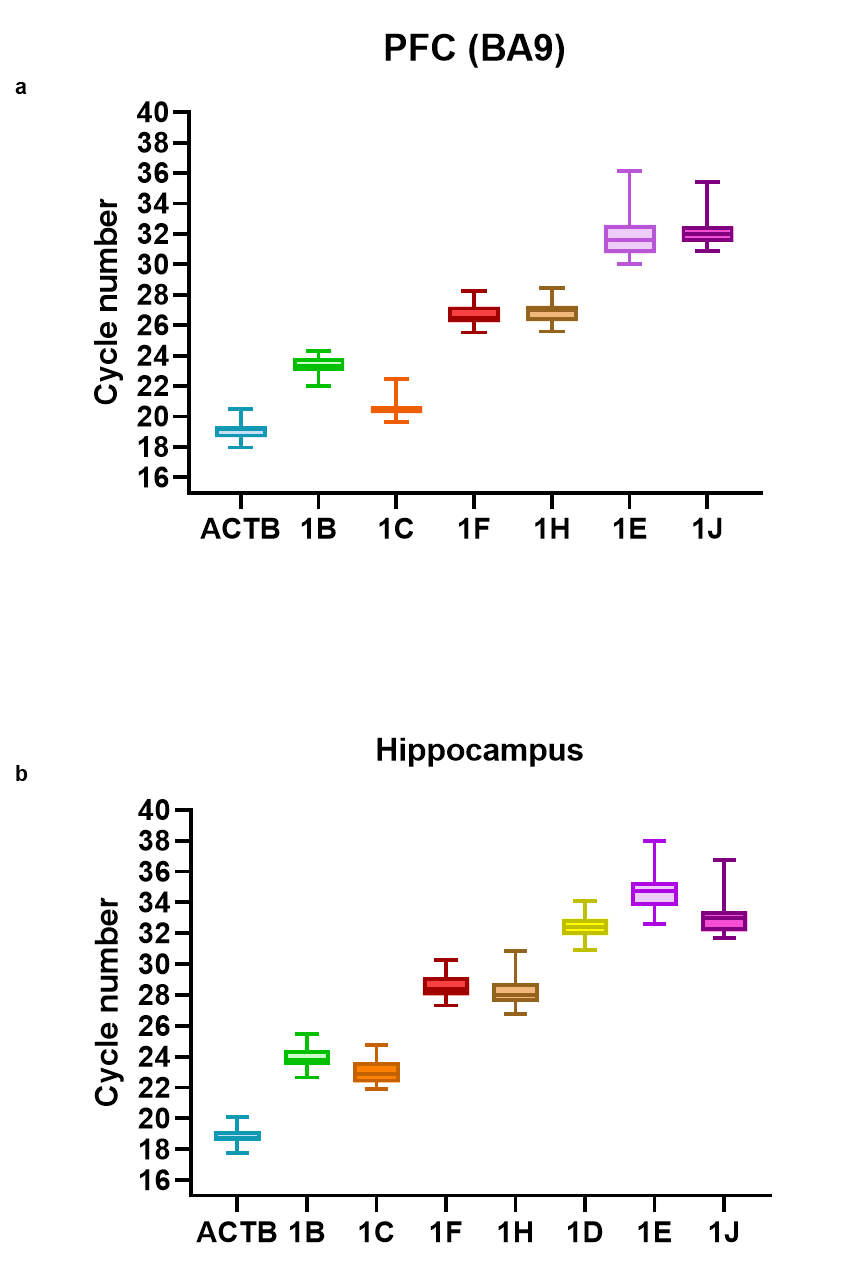
**Figure S2. Relative expression of GR exon-1 transcript variants**

**Figure S2.**

Box-and-whisker plot indicating range of Ct values of GR-1 transcript variants and the normalizer β-Actin (ACTB). **a.** In the PFC and **b.** in the hippocampus. Values are taken as averages of 24 healthy controls and the whiskers represent standard deviation of n samples (n = 24).

**Table S4. Demographic Characteristics of Teenage Non-psychiatric Control and Suicide Subjects**

| **Patient No./**  **Sex/Age,y/Race** | **PMI (h)** | **Brain pH** | **RIN (PFC)** | **RIN (Hippocampus)** | **Cause of Death** | **Psychotropic Drugs**  **(at the time of death)** | **Psychiatric Diagnosis** |
| --- | --- | --- | --- | --- | --- | --- | --- |
|  |  |  |  |  |  |  |  |
| **NON PSYCHIATRIC CONTROL GROUP^*^** |  |  |  |  |  |  |  |
|  |  |  |  |  |  |  |  |
| 1/M/19/B | 6 | 6.14 | 7.4 | 5.1 | GSW | None | Normal |
| 2/M/16/B | 6 | 6.54 | 6 | 5.9 | GSW | None | Normal |
| 3/M/16/B | 8 | 5.64 | 7.2 | 4.8 | GSW | None | Normal |
| 4/M/19/B | 12 | 5.9 | 7.3 | 6.1 | GSW | None | Normal |
| 5/M/13/W | NA | 5.19 |  |  | Accident | None | Normal |
| 6/M/17/B | 11 | 6.52 | 8 | 6.1 | GSW | None | Normal |
| 7/M/16/W | 10 | 5.42 | 8.3 | 4.8 | Stabbing | None | Normal |
| 8/M/17/B | 10 | 5.9 | 8.8 | 6.1 | GSW | None | Normal |
| 9/M/13/B | 22 | 6.07 | 9.3 | 5.3 | GSW | None | Normal |
| 10/M/14/B | 18 | 5.73 | 9.1 | 5.9 | GSW | None | Normal |
| 11/M/18/B | 27 | 6.09 | 8.7 | 5.2 | Drowning | None | Normal |
| 12/M/16/W | 21 | 5.97 | 8.3 | 5.8 | Accidental hanging | None | Normal |
| 13/F/18/W | 35 | 5.99 | 8.9 | 4.9 | Multiple injuries | None | Normal |
| 14/F/17/B | 26 | 6.23 | 10 | 5.4 | Multiple injuries | None | Normal |
| 15/F/19/W | 30 | 6.2 | 7.7 | 5.5 | Cardiac arrhythmia | None | Normal |
| 16/F/18/B | 16 | 6.6 | 8.6 | 6.6 | MVA | None | Normal |
| 17/M/13/B | 20 | 6.6 | 10 | 6.8 | Drowning | None | Normal |
| 18/M/19/B | 16 | 6.6 | 9.1 | 6.2 | Congenital heart disease | None | Normal |
| 19/F/16/W | 24 | 6.71 | 8.7 | 7.3 | Myocarditis | None | Normal |
| 20/M/15/W | 16 | 6.5 | 8.7 | 7.3 | Cardiac arrhythmia | None | Normal |
| 21/M/15/W | 21 | 6.37 | 5.9 | 5.4 | PE/DVT | None | Normal |
| 22/F/16/W | 20 | 6.89 | 10 | 5.7 | MVA | None | Normal |
| 23/F/13/B | 23 | 6 | 8.3 | 7.4 | Accidental Hanging | None | Normal |
| 24/M/18/W | 19 | 5.80 |  |  | Complications of Morbid Obesity | None | Normal |
| **SUICIDE GROUP^+^** |  |  |  |  |  |  |  |
|  |  |  |  |  |  |  |  |
| 1/F/15/W | 7 | 5.48 | 6.4 | 5.3 | GSW | Ethanol | Alcohol abuse |
| 2/M/20/W | 32 | 6.41 | 8 | 6.1 | Hanging | Ethanol | Alcohol abuse |
| 3/M/12/B | 10 | 5.91 | 6.9 | 6 | Hanging | None | Major depression |
| 4/M/15/W | 11 | 5.33 | 8.1 | 7 | Asphyxia | None | Major depression |
| 5/F/15/W | 17 | 5.58 | 8.6 | 5.4 | Drug overdose | Imipramine, Desipramine | Major depression, Hyperactivity attention deficit disorder |
| 6/M/15/W | 27 | 6.08 | 9.2 | 6.2 | GSW | None | Adjustment disorder, Major depression |
| 7/M/18/W | 17 | 6.3 | 9.5 | 4.9 | Hanging | None | Major depression - single episode |
| 8/M/19/W | 18 | 6.2 | 9.1 | 7 | CO intoxication | Ethanol | Major depression, Ethanol abuse, Polysubstance abuse |
| 9/F/15/W | 20 | 6.59 | 8.1 | 5.2 | Hanging | None | Major depression - single episode, Ethanol abuse |
| 10/M/17/W | 23 | 6.66 | 7.3 | 5.2 | Hanging | Ethanol | Major depression - single episode, Ethanol abuse |
| 11/M/13/W | 18 | 6 | 6.7 | 5.1 | Hanging | Ritalin | Hyperactivity attention deficit disorder |
| 12/F/17/W | 25 | 5.55 | 6.5 | 5.9 | Drug overdose | None | Adjustment disorder |
| 13/F/16/W | 33 | 6.61 | 8.7 | 5.9 | GSW | None | Adjustment disorder |
| 14/M/16/W | 24 | 6.81 | 9.4 | 6.2 | Hanging | None | Adjustment, Conduct disorders |
| 15/F/15/W | 21 | 6.48 | 8.6 | 7.1 | Hanging | None | Adjustment disorder with depressed mood |
| 16/F/15/W | 20 | 6.1 |  |  | Hanging | None | Borderline personality disorder |
| 17/M/19/W | 15 | 6.9 | 9.6 | 4.9 | GSW to chest | Fluoxetine | Dissociative disorder, Substance abuse (kind unclear), PTSD |
| 18/F/15/W | 11 | 6.44 | 8.3 | 4.8 | Drug overdose | None | No mental disorder |
| 19/M/17/A | 7 | 5.9 | 4.7 | 5 | GSW | Ethanol | No mental disorder |
| 20/M/16/H | 20 | 6.17 | 9.1 | 6.1 | Hanging | None | No mental disorder |
| 21/F/16/W | 18 | 6.31 | 8.7 | 5.7 | GSW | Amitriptyline | No mental disorder |
| 22/M/15/W | 16 | 6.18 | 9.7 | 7.4 | Hanging | None | No mental disorder |
| 23/M/14/B | 22 | 6.44 | 9.8 | 6.4 | Hanging | None | No mental disorder |
| 24/F/17/W | 24 | 6.49 | 8.3 | 4.9 | Diphenhydramine overdose | Citalopram | Dx (not enough info) |
|  |  |  |  |  |  |  |  |

Abbreviations: M, male; F, female; B, black; W, white; A, Asian; H, Hispanic; ACSVD, atherosclerotic cardiovascular disease; GSW, gunshot wound; MVA, motor vehicle accident; NA, not available; PE/DVT, pulmonary embolism, deep vein thrombosis; PMI, postmortem interval

**Table S5: Uncorrected and FDR-adjusted p-values**

|  | p-values | Corrected p-values (FDR) |
| --- | --- | --- |
| GRP_PFC | 0.003 | 0.0037 |
| GR1B_PFC | 0.009 | 0.009 |
| GR1C_PFC | 0.0003 | 0.00075 |
| GR1F_PFC | 0.002 | 0.0033 |
| GR1H_PFC | 0.00005 | 0.00025 |
|  |  |  |
| GRP_HIPPO | 0.75 | 0.8 |
| GR1B_HIPPO | 0.38 | 0.8 |
| GR1C_HIPPO | 0.66 | 0.8 |
| GR1F_HIPPO | 0.42 | 0.8 |
| GR1H_HIPPO | 0.8 | 0.8 |
|  |  |  |
| TET1_PFC | 0.03 | 0.045 |
| TET2_PFC | 0.02 | 0.045 |
| TET3_PFC | 0.52 | NA |
| DNMT1_PFC | 0.02 | 0.045 |
| DNMT3A_PFC | 0.02 | 0.045 |
| DNMT3B_PFC | 0.11 | NA |
| GADD45A_PFC | 0.43 | NA |
| GADD45B_PFC | 0.03 | 0.045 |
| GADD45G_PFC | 0.81 | NA |
|  |  |  |
| TET1_HIPPO | 0.08 | 0.24 |
| TET2_HIPPO | 0.246 | 0.51 |
| TET3_HIPPO | 0.34 | 0.51 |
| DNMT1_HIPPO | 0.02 | 0.51 |
| DNMT3A_HIPPO | 0.29 | 0.18 |
| DNMT3B_HIPPO | 0.48 | 0.54 |
| GADD45A_HIPPO | 0.44 | 0.54 |
| GADD45B_HIPPO | 0.04 | 0.18 |
| GADD45G_HIPPO | 0.6 | 0.6 |
|  |  |  |
| R1MC_PFC | 0.01 | 0.013 |
| R1HMC_PFC | 0.86 | 0.910 |
| R2MC_PFC | 0.25 | 0.392 |
| R2HMC_PFC | 0.19 | 0.348 |
| R3MC_PFC | < 0.001 | 0.0003 |
| R3HMC_PFC | < 0.001 | 0.0003 |
| R4MC_PFC | 0.78 | 0.910 |
| R4HMC_PFC | 0.34 | 0.467 |
| R5MC_PFC | 0.002 | 0.005 |
| R5HMC_PFC | < 0.001 | 0.0003 |
| R6MC_PFC | .91 | .91 |
|  |  |  |
| FKBP5_PFC | .008 | .013 |
| FKBP5_HIPPO | .02 | .02 |
| FKBP5_MC | .001 | .004 |
| FKBP5_HMC | .01 | .013 |
